# Oral Health Management and Rehabilitation for Patients with Oral Cancer: A Narrative Review

**DOI:** 10.3390/healthcare10050960

**Published:** 2022-05-23

**Authors:** Yuhei Matsuda, Ruwan D. Jayasinghe, Hui Zhong, Shinichi Arakawa, Takahiro Kanno

**Affiliations:** 1 Department of Lifetime Oral Health Care Sciences, Graduate School of Medical and Dental Sciences, Tokyo Medical and Dental University, Tokyo 113-8501, Japan; heibla966@gmail.com (H.Z.); shinperi@tmd.ac.jp (S.A.); 2 Department of Oral and Maxillofacial Surgery, Shimane University Faculty of Medicine, Izumo 693-8501, Japan; tkanno@med.shimane-u.ac.jp; 3 Center for Research in Oral Cancer, Department of Oral Medicine and Periodontology, Faculty of Dental Sciences, University of Peradeniya, Getambe 20400, Sri Lanka; ruwanduminda@yahoo.com

**Keywords:** oral cancer, oral health management, oral function management, oral hygiene management, oral care, rehabilitation

## Abstract

Surgery is the current first choice for oral cancer treatment. Intensity-modulated radiation therapy, molecular targeted drugs, and immune checkpoint inhibitors are still used as adjuvant therapy for advanced cancer. In addition, postoperative rehabilitation and multidisciplinary treatment have also been developed in recent years. Multidisciplinary team approaches and supportive care in oral cancer treatment reportedly shorten the time to treatment and improve outcomes. Although there is enough evidence confirming the role of oral and maxillofacial surgeons, dentists, and dental hygienists in supportive care in oral cancer treatment, there are very few systematic studies. In particular, oral health management is a concept that encompasses oral function management, oral hygiene management, and oral care during oral cancer treatment. We provide a narrative review focusing on oral health management from a multidisciplinary and supportive care perspective, applicable in oral cancer treatment.

## 1. Introduction

**Figure 1:**
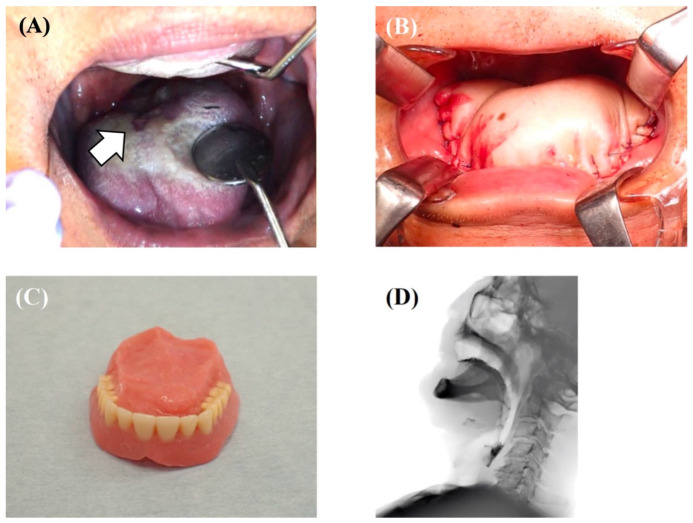
A case of tongue squamous cell carcinoma (T4aN3bM0, Stage IVB) treated with a tracheotomy, tumor resection, modified radical neck dissection (Type III), and pectoralis major musculocutaneous flap reconstruction; the patient recovered to be able to eat with palatal augmentation prosthesis. After completing chemoradiotherapy, the patient takes almost all of their nutrition orally and does well, with no recurrence or metastasis. (**A**) Mirror view of the primary tumor on the dorsum of the tongue. (**B**) Intraoperative photo of tongue reconstruction by pectoralis major musculocutaneous flap. (**C**) Palatal augmentation prosthesis. (**D**) Findings of videofluoroscopic examination of swallowing where the patient was able to swallow jelly with palatal augmentation prosthesis.

Oral and oropharyngeal cancer is the sixth most common cancer worldwide [1]. The incidence of oral cancer is particularly serious in South Central Asia and includes three common cancers [2]. It has been reported that the number of patients is increasing due to increased exposure to risk factors, especially in Asia [3]. Much of the modern knowledge about oral cancer treatment comes from the 19th and 20th centuries; it can be traced back to 1841 when Theodor Kocher and his colleagues operated on 120 patients with oral cancer [4]. Around 1905–1906, cervical dissection was proposed as an extended resection for oral cancer. In 1963, conservative cervical dissection was developed to preserve the accessory nerves and other parts of the body [4,5]. In addition, the development of reconstructive surgery has also contributed greatly to the improvement of patients’ quality of life (QoL), and surgery under computer simulation is becoming more common [6]. Currently, surgery is still the first choice for oral cancer treatment in both the elderly and the young [7]. Still, intensity-modulated radiation therapy, molecular targeted drugs, and immune checkpoint inhibitors are now used as adjuvant therapy for advanced cancer [8,9,10]. However, it is not only the treatment but also postoperative rehabilitation and multidisciplinary treatment that have been developed in recent years. In fact, it is not a single disability after oral cancer treatment, but rather multiple disabilities (dysphagia, dysarthria, esthetic disorders, and psychosocial disorders) that can significantly impair QoL, thus requiring multidisciplinary intervention [11]. The fact that the suicide rate among oral cancer patients, or those who have undergone oral cancer treatment, is the highest among all cancers is another indication of the intensity and difficulty of oral cancer treatment [12,13]. Multidisciplinary team approaches and supportive care have been reported to shorten the time to treatment and improve outcomes. A review article by Licitra et al. stated that the core team for oral cancer requires intervention from 20 professions and that the extended team requires intervention from 32 professions, including oral management specialists such as oral and maxillofacial surgeons, dentists, and dental hygienists [14]. In fact, even patients who have undergone intense oral cancer treatment can return to society with an appropriate team approach to oral health management and rehabilitation (Figure 1A–D). However, although scattered evidence exists on the role of oral management specialists in supportive care in oral cancer treatment, there are few systematic studies. In particular, there are very few studies on oral health management as a concept that encompasses oral function management, oral hygiene management, and oral care in oral cancer treatment. In addition, there are no established follow-up programs for post-treatment oral cancer patients in daily clinical practice [15]. Therefore, the purpose of this article was to provide a narrative review focusing on oral health management from a multidisciplinary and supportive care perspective in oral cancer treatment.

## 2. Oral Health Management in Oral Cancer Patients

**Figure 2:**
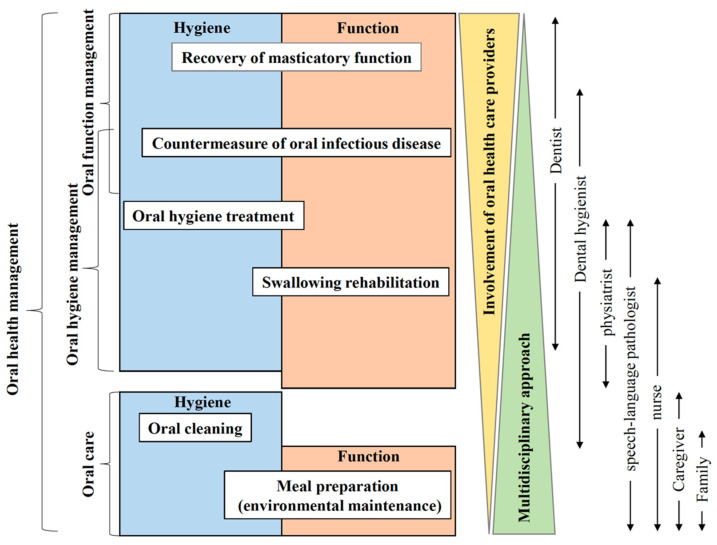
Oral health management in oral cancer treatment.

**Figure 3:**
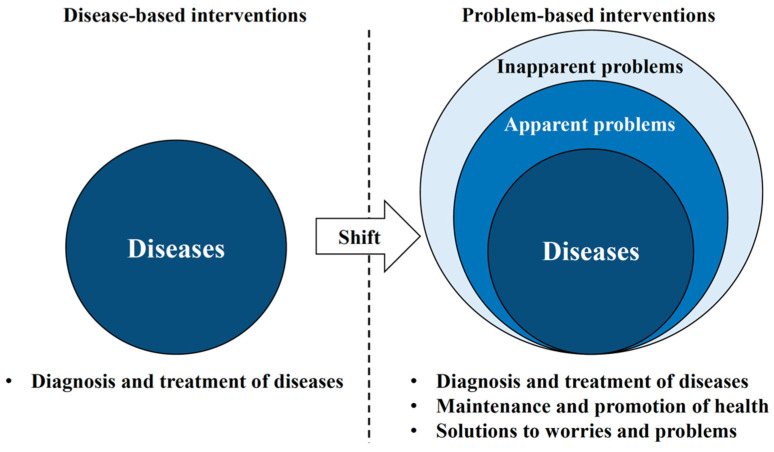
Oral health management is a paradigm shift in oral health care.

Oral health management is defined as interventions for comprehensive oral-related factors (Figure 2). According to a review by Wong et al., systemic condition (body mass index, cognitive function, and comorbidities), oral health status (presence of dental caries and periodontal disease), general oral problems (mucosal, dental, periodontal, and temporomandibular joint problems, denture problems, masticatory function, and functional tooth units), oral health-related quality of life (QoL), non-modifiable factors (age, sex, race, and educational level), and modifiable factors (dental service attendance and service accessibility, dependent individuals, medications, health insurance, and income) have been identified as related factors [16,17]. In other words, oral health management is a paradigm shift for dental practitioners because it requires a shift from a disease-based approach to a problem-based approach (Figure 3). Therefore, based on human needs, the conceptual model is used in the dental hygiene process as an indicator of concepts, including oral hygiene management and oral function management, to identify how patient problems may guide intervention [18].

### 2.1. Oral Function Management in Oral Cancer Patients

In Japan, oral function management in oral cancer patients is referred to as “perioperative oral management.” Since its inclusion in the national health insurance system in 2012, clinical and research activities have become popular [19]. In recent years, the definition of an oral function has become easier to understand by referring to the oral hypofunction assessment (poor oral hygiene, oral dryness, reduced occlusal force, decreased tongue–lip motor function, decreased tongue pressure, decreased masticatory function, and deterioration of swallowing function) proposed by the Japanese Society of Gerodontology [20].

#### 2.1.1. Poor Oral Hygiene

**Figure 4:**
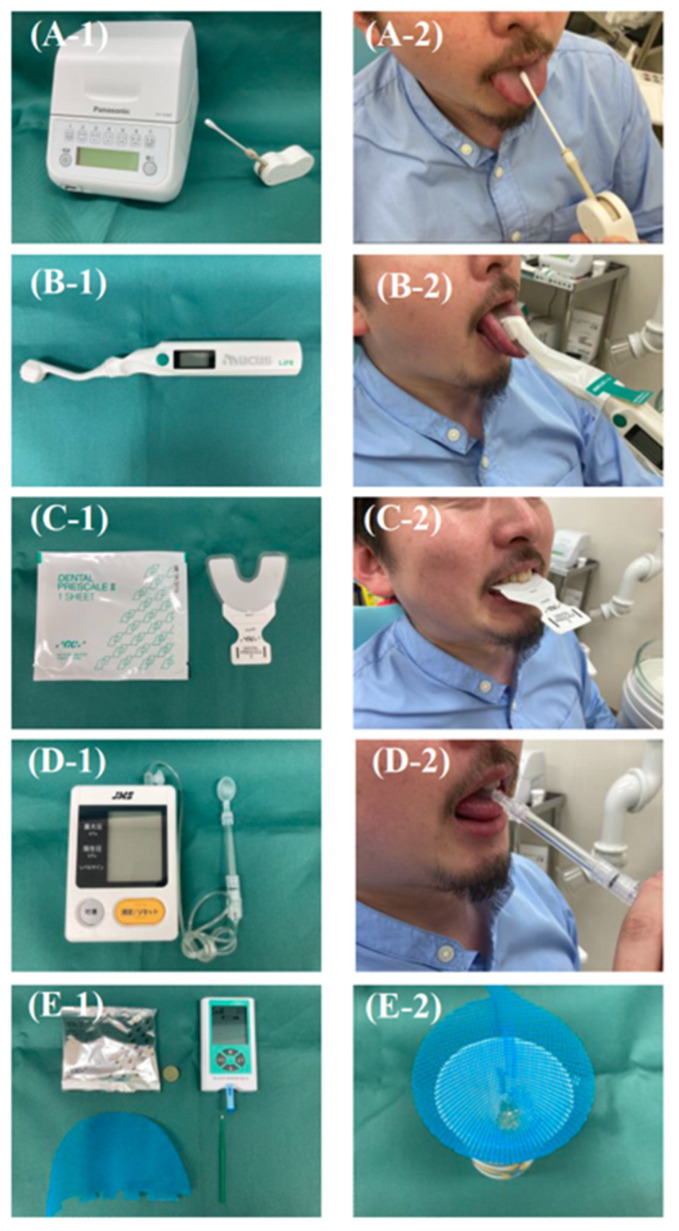
Testing instruments for oral function assessment and their use. (**A-1**) Bacterial counter. (**A-2**) Specimen being collected from the dorsum of the tongue. (**B-1**) Oral moisture checker. (**B-2**) Measuring oral moisture on the dorsal surface of the tongue. (**C-1**) Dental prescale. (**C-2**) Measurement of occlusal force using dental prescale. (**D-1**) JMS tongue pressure measuring instrument. (**D-2**) Measurement of tongue pressure using a probe. (**E-1**) Gluco Sensor GS-II. (**E-2**) After chewing and filtering gummy jelly.

Poor oral hygiene is defined as a higher than a normal number of microorganisms in the oral cavity, especially in oral cancer patients, which means a higher risk of surgical site infection and aspiration pneumonia (Figure 4(A-1,A-2)) [20,21,22]. Poor oral hygiene is described in the section Oral Hygiene Management in Oral Cancer Patients and is described below.

#### 2.1.2. Oral Dryness

Oral dryness refers to a condition in which the oral cavity is drier than normal or in which there are subjective symptoms associated with a feeling of dryness in the oral cavity. This condition is often reported during follow-up to chemoradiotherapy, especially in oral cancer treatment (Figure 4(B-1,B-2)) [20,23]. The cause of xerostomia in oral cancer treatment is mainly due to the effects of salivary gland resection and organic changes in salivary gland tissue caused by radiation and chemotherapy [24]. A cross-sectional study investigating quality of life using the EORTC-QLQ H&N35 reported that reconstructive surgery decreases dry mouth, but in general, resection of the salivary glands causes dry mouth [25]. For radiation therapy (RT), 89 intensity-modulated radiotherapy (IMRT) is used more often than RT, and a meta-analysis reported that the IMRT group had a better QoL for dry mouth (pooled standardized mean difference (SMD) = −0.60, 95% confidence interval −0.97 to −0.24, *p* = 0.001) [26]. As Wolff et al. pointed out in their systematic review, chemotherapy-induced dry mouth is caused by multiple drugs, including cisplatin [27]. Measures to combat dry mouth include the substitution or discontinuation of medications whenever possible, topical oral or systemic administration of sialogens, administration of substitute saliva, and the use of electrical stimulators, with substitute saliva being important in oral health management. However, at present, there are no reports of substitute saliva with a high level of evidence, even in review articles by the Cochrane Collaboration [28]. Thus, the empirical selection is necessary, relying on the patient’s feelings of use.

#### 2.1.3. Reduced Occlusal Force

Reduced occlusal force is a condition of decreased occlusal force with natural teeth or dentures, which can be caused by a decrease in the number of teeth or the removal of masticatory muscles, especially in oral cancer treatment that involves maxilla and mandible resection (Figure 4(C-1,C-2)) [20,29]. In patients with a resected maxilla and mandible for oral cancer treatment, reduced occlusal force is affected by Eichner C’s occlusal support classification, with large individual differences [30]. There was no difference in the occlusal forces of the incisors and first molars in patients who underwent mandibulectomy compared to those in healthy subjects. Additionally, occlusal forces were restored with the use of a bone-anchored device for wide edentulous areas [31,32]. By contrast, in maxillectomy patients, the masticatory function can be restored by prosthesis, but occlusal force exists as a factor independent of masticatory function and may not be restored [33]. It has also been reported that occlusal function may not be optimally controlled in maxillofacial reconstruction patients. Zheng et al. reported that maximal muscle strength decreases in maxillofacial reconstruction patients due to abnormalities of the masticatory muscles, where the magnitude of muscle strength fluctuates during the rehabilitation period. They also pointed out that the decrease in masticatory muscle activity on the resected side may cause non-physiological oral biomechanical responses and alter muscle activity to stabilize the reconstructed mandible [29]. To the best of our knowledge, there is no intervention method for strengthening occlusal force in patients with oral cancer, except for prosthetic treatment, but it is hoped that muscle strengthening methods will be developed in the future.

#### 2.1.4. Tongue–Lip Motor Function

Decreased tongue–lip motor function is defined as a decrease in the speed and dexterity of tongue and lip motor skills and has been reported as a sequela of tongue resection, especially in oral cancer treatment [20,34]. A prospective cohort study of 123 patients with oral cancer, in which tongue strength, mobility, and sensory function were measured 4 weeks before treatment, 4–6 weeks after treatment, and 6 months, 1 year, and 5 years after treatment reported that tongue function deteriorated significantly after oral tumor treatment, with poor prosthetics being an aggravating factor [35]. Patients who have undergone having more than half of their tongue volume resected require reconstructive surgery with a flap to restore tongue volume and function. However, it has been reported that there is no difference in speech intelligibility or tongue mobility between types of flaps (comparing a free forearm flap with an anterolateral thigh flap) [34,36]. In particular, a study that evaluated tongue function in 238 oral cancer patients, reconstructed with an anterolateral thigh flap, reported that individually designed anterolateral thigh flaps were significantly better than commonly designed anterolateral thigh flaps in maintaining not only oral volume, but also tongue mobility, speech intelligibility, and swallowing ability at 6 months, suggesting that functional reconstruction may be possible with further surgical improvements [37]. A study investigating tongue function using functional magnetic resonance imaging (MRI) of 19 subjects, including healthy subjects and oral cancer patients, reported that different groups of tongue muscles tend to function during speech, especially the floor-of-the-mouth muscles, which have a unique pattern of supporting the tongue and helping it to rotate, suggesting that strength training of the floor-of-the-mouth muscles may be effective in improving tongue–lip motor function [38]. In contrast, a study investigating the profile of tongue function in 47 patients with oral cancer who underwent maxillectomy reported that oral diadochokinesis and tongue pressure improved significantly in patients who wore prosthetic devices, suggesting the need to consider prosthetic approaches for patients with decreased tongue–lip motor function [39]. In addition, a prospective cohort interventional study investigating the effects of an 8-week progressive lingual exercise program on 10 healthy elderly subjects reported that intraoral MRI increased tongue volume by an average of 5.1%, which may apply to improving tongue–lip motor function in oral cancer patients [40].

#### 2.1.5. Tongue Pressure

Decreased tongue pressure in patients with oral cancer is defined as a decrease in pressure between the tongue and the palate and food due to organic or functional loss of the muscle groups that move the tongue, which can be caused by multiple factors, including surgical treatment and radiation chemotherapy (Figure 4(D-1,D-2)) [41,42]. Hasegawa et al. reported that tongue pressure in 57 patients who underwent surgery for oral cancers and head and neck cancers decreased significantly in the first 1–2 weeks after surgery and recovered over time. In addition, changes in tongue pressure were significantly associated with cancer stage, radiotherapy, and reconstruction, and receiver operating characteristic analysis revealed that a tongue pressure of 15 kPa was the cutoff value for detecting postoperative dysphagia [43]. As a method of improving tongue pressure, flap and scar revision as a surgical treatment has been reported to reduce postoperative swallowing dysfunction in oral cancer patients and is suggested as an intervention method [44]. As a non-invasive intervention, the patient was instructed to press the tongue as hard as possible against the palate with the mouth closed for 10 s of exercise, followed by 10 s of rest. One set consisted of five consecutive exercise and rest periods. As a result of performing two sets a day for one month in elderly patients, the researchers reported that they could restore tongue strength (especially the function of the suprahyoid muscle), which may apply to oral cancer patients [45].

#### 2.1.6. Masticatory Function

A decreased masticatory function is defined as a decrease in the ability to chew food and mix it with saliva to form a food mass, due to decreased occlusal force and tongue mobility (Figure 4(E-1,E-2)) [18]. An observational study that measured masticatory function one year after surgery, in 45 patients with oral cancer, indicated that surgery has a significant negative impact on masticatory function and that radiotherapy is an aggravating factor [46]. Several papers have pointed out that factors affecting masticatory function in oral cancer treatment have a strong influence, in the following order: the extent of hard palate defect > status of posterior mandibular teeth > maximum occlusal force > mouth-opening distance [30,47,48,49]. Oral and head and neck cancers frequently have complications related to malnutrition. Although it has been reported that impaired masticatory function is not a causative factor, it is a problem that must be addressed because poor masticatory function has been reported to have a significant impact on patients’ QoL [50,51]. Prosthetic treatment is the first choice to restore masticatory function, especially for the use of bone-anchored devices for wide edentulous areas, and cooperation between oral surgeons and prosthodontists is important [52]. Barbu et al. reported that a bone-anchored device for a wide edentulous area showed a significant improvement in prosthetic and patient satisfaction compared to non-implant-retained prostheses [53]. In addition, it was reported that the function of patients with a bone-anchored device for wide edentulous areas was significantly and substantially improved (maximum occlusal force ranged from 77.5% to 365 N, 371%). The assessment of masticatory function by a color chart of gummy chewing was also significantly improved by about 208% [54]. In addition, a 5-year prospective study of a bone-anchored device for a wide edentulous area in 56 oral cancer patients noted a functional benefit of implant placement during resection surgery. In contrast, a retrospective study measuring occlusal forces using the T-Scan III in 13 patients with oral cancer, who underwent microvascular free fibular flap reconstruction and prosthetic treatment with a bone-anchored device for a wide edentulous area, suggested that the crown-to-implant ratio did not significantly correlate with maximum occlusal forces or peri-implant fibrous resorption, and that increasing the length of the fibrous flap of the reconstructed mandibular implant-supported prosthesis reduced occlusal forces, suggesting further improvement in the future [55].

#### 2.1.7. Swallowing Function

Decreased swallowing function in patients with oral cancer is defined as the presence of functional impairment as a precursor to significant disability, either due to cancer itself or the effects of treatment [20]. For the swallowing function, the details are described in Section 3.1.

### 2.2. Oral Hygiene Management in Oral Cancer Patients

Oral hygiene management has been shown to prevent changes in oral cancer treatment schedules and increase the likelihood of completing the protocol [56,57]. The purpose of oral hygiene management for patients with oral cancer varies depending on the treatment modality. In any case, the role of the dental professional continues from before the diagnosis of cancer through survivorship. It includes oral screening and maintenance, managing common oral complications such as mucositis, pain, infection, salivary insufficiency, taste disorders, and tooth decay, and complex issues such as soft tissue fibrosis and osteonecrosis of the jaw, and dysphagia [58]. However, the Eilers Oral Assessment Guide and other indices have been adapted to assess the oral cavity of oral cancer patients, and the reliability and validity of the assessments have been verified in nurses and dental hygienists. Still, their widespread use has been limited [59]. In addition, several papers have pointed out that there is a general lack of consistency in how, when, and with whom oral cancer patients receive oral health education, and this paper systematically reviews the limited evidence, despite the variation in intervention methods [60,61]. Thus, although there is no uniform intervention method, a review of oral hygiene management for oral cancer patients by purpose and effect is presented below.

#### 2.2.1. Dental Caries, Periodontal Disease, and Oral Candidiasis

**Figure 5:**
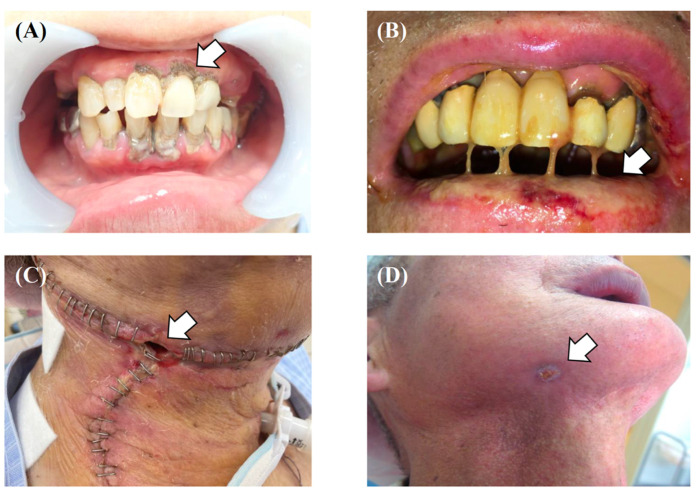
(**A**) Intraoral view with a large amount of dental calculus. (**B**) Grade 3 oral mucositis caused by chemoradiotherapy. (**C**) Wound healing failure in the neck caused by surgical site infection. (**D**) Exodontic fistula caused by osteoradionecrosis in stage 3.

When the tongue is resected for oral cancer treatment, the bacterial balance and pathogenicity in saliva are significantly altered. *Streptococcus salivarius*, *Prevotella melaninogenica*, and *Prevotella histicola* are significantly decreased after surgery. In contrast, *Lautropia mirabilis*, *Neisseria flava*, *Streptococcus sanguinis*, and *Fusobacterium nucleatum* are increased significantly after surgery [21]. In particular, the oral environment, altered by surgery and xerostomia and decreased saliva secretion due to the effects of radiotherapy, makes radiation caries more likely to occur, which are not easy to prevent, even with intensive oral cleaning [62]. As a countermeasure against radiation caries, it has been confirmed that the application of fluoride (topical fluoride with 10% CPP–ACP paste) significantly reduces the incidence of dental caries in patients treated with oral cancer radiotherapy, since the amount of endogenous Ca^2+^ and PO43−, which promotes remineralization, is reduced in saliva due to decreased saliva volume [63]. Since the caries-preventive effect of fluoride (1100 ppm formulation) toothpaste was reported in a randomized controlled trial for caries control in 57 patients treated with head and neck radiotherapy, fluoride application in patients treated for oral cancer is also recommended [64]. On the other hand, a review article on the relationship between cancer treatment and periodontal disease indicates that periodontal disease may worsen during cancer treatment, causing oral pain, infection, and systemic infections, and may increase morbidity and mortality, especially in febrile neutropenic cancer patients (Figure 5A) [65]. In addition, patients undergoing head and neck radiotherapy are known to be more susceptible to dental caries and periodontal disease, as well as opportunistic infections of the oral mucosa, such as *Candida albicans* [66]. Regarding periodontal disease, a study in radiotherapy patients with upper respiratory tract cancer reported that it could be improved by basic periodontal treatment [67]. Therefore, although there are no studies that provide clear criteria, it is thought that initial treatment for dental caries, periodontal disease, and oral bacteria should be completed as early as possible before oral cancer treatment.

#### 2.2.2. Oral Mucositis

Oral mucositis has been reported to develop rapidly after chemotherapy, radiation therapy, and combination therapy (Figure 5B) [68]. Bacterial infection is known to be an aggravating factor of oral mucositis, and it has been reported that completion of an oral hygiene protocol can mildly reduce the grade of oral mucositis, as defined by the National Cancer Institute—Common Terminology Criteria for Adverse Events (NCI-CTCAE) [69,70,71]. In addition, in a systematic review conducted by the mucositis study group of the Multinational Association of Supportive Care in Cancer (MASCC) and the International Society of Oral Oncology (ISOO), a society promoting supportive care to control side effects and complications associated with cancer treatment, the implementation of an oral health management protocol was proposed as beneficial (evidence level III) [72]. However, there is no unified view on the content of oral management protocols for oral mucositis, and we still have to rely on empirical interventions in which the opinions of experts are adopted.

#### 2.2.3. Surgical Site Infection

Tongue resection causes a change in the composition of the salivary microbiota, characterized by an increase in dental plaque-derived bacterial species, including periodontal bacteria, which increases the risk of developing surgical site infection (SSI), especially by Gram-negative anaerobic rods (Figure 5C) [21]. In patients with SSI, suture failure can lead to the formation of a pathway between the oral cavity and neck, and increased drainage, leading to prolonged hospital stay due to a renewed systemic inflammatory response [73,74]. Recently, a multicenter randomized controlled trial examining the efficacy of topical tetracycline ointment, in addition to basic oral cleaning, reported that local administration of tetracycline 48 h after surgery could reduce the incidence of SSI after oral cancer surgery [75,76]. Therefore, in addition to basic oral cleaning as a measure against SSI, the application of tetracycline should be considered as an option for patients with a poor oral environment.

#### 2.2.4. Osteoradionecrosis of Jaw

Osteoradionecrosis of the jaw (ORNJ), caused by the radiation field entering the oral cavity, tends to develop close to the primary tumor site and is likely triggered by surgical procedures such as tooth extraction, within 6 months of the irradiation (Figure 5D) [77,78]. Gender, dentition, and chemotherapy do not affect the development of ORNJ; a paper that analyzed risk factors for the development of ORNJ in 776 head and neck cancer patients that received intensity-modulated radiation therapy (IMRT), identified the location of the tumor in the oral cavity, radical radiation therapy or not, and previous surgical treatment of the maxilla and mandible as risk factors [79,80]. However, in a case-control study of risk factors for the development of ORNJ in head and neck cancer patients, the use of chlorhexidine mouthwash (1.28-fold) and scaling within 2 weeks before radiotherapy (2.43-fold) were reported as risk factors for the development of ORNJ. Therefore, careful prescription of mouthwash and appropriate timing of scaling should be considered [81].

### 2.3. Oral Care (Oral Health Care Provided by Non-Dental Professionals)

Oral care (cleaning and exercising the mouth by a non-dental professional) requires a different approach than direct intervention by a medical professional.

#### 2.3.1. Self-Care by the Patient

Self-care by the patient and the dental care provider is important for oral-related care after oral cancer treatment. Although the method of self-care after oral cancer treatment can be complex and difficult, empowering patients to develop effective self-care skills (hygiene behavior, functional training, and self-examination) is believed to result in a favorable outcome [82]. It is also important to provide information to improve oral-related literacy when providing self-care skills and to observe patients’ attitudes and, if necessary, approach oral health-related self-efficacy to encourage behavior change [83,84,85].

#### 2.3.2. Oral Care by the Patient’s Family

Educating family members also plays an important role in providing patients with continuous oral care [86]. In other words, oral care requires educating all stakeholders, including healthcare providers [87]. Delays in seeking care for patients are associated with social and familial interactions with cancer [88]. A report examining 125 couples with head and neck cancers, including oral cancer, reported that spouses focus primarily on maintaining the patient’s weight and encouraging hydration. Oral care may be an afterthought [89]. In fact, an intervention study that trained 30 family caregivers of cancer patients in basic skills, including oral care, reported that patients’ QoL (physical, emotional, and social functioning) and pain improved [90]. However, patients with terminal-stage oral cancer often experience changes in their oral status, which may require more frequent changes in intervention methods [91]. Therefore, oral care programs that encourage spouses in maximizing positive social control may enhance patients’ mood during treatment and improve QoL [89].

#### 2.3.3. Oral Care by Medical Staff

Although multidisciplinary cooperation is important in oral cancer treatment, it goes without saying that dental care providers are a part of this. A different professional approach is needed when patients are discharged from the acute phase of treatment and return to the local community [14]. In particular, interventions by primary care physicians, visiting nurses, caregivers, and, if necessary, family dentists and dental hygienists are important, and measures must be taken to deal with late side effects that persist months to a year or more after the end of treatment, which are often forgotten [92,93]. Therefore, dentists and dental hygienists in acute care hospitals need to pay attention to teaching non-dental healthcare providers.

## 3. Oral Rehabilitation

The effects of oral cancer treatment may include changes in oral anatomy, loss of teeth, loss of anatomical structures such as the tongue and soft palate, changes in muscle attachment and muscle balance, loss of lip function, presence of bulging flaps, loss or changes in sensation, development of the semicircular canal, and changes in the appearance of the oral face [94]. This section reviews oral disorders that were not mentioned in the oral health care section above (Section 2.1, Section 2.2 and Section 2.3), placing them in the context of oral rehabilitation.

### 3.1. Oropharyngeal Dysphagia (Difficulty Swallowing)

**Figure 6:**
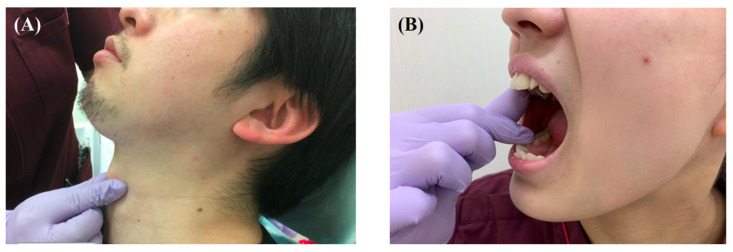
Functional swallowing exercises for oral cancer patients. (**A**) Performing the Mendelssohn maneuver. (**B**) Manual opening training.

This review focuses on dysphagia in the pharyngeal and esophageal phases of the five-phase model of swallowing, or in the stage 2 transport, pharyngeal bolus aggregation, and swallowing phases of the process model [95,96]. In surgery, it has been reported that high tumor stage, highly invasive surgery, and reconstructive surgery with a free flap are independently associated with poor postoperative oral intake [97,98]. In addition, postoperative chemoradiation is a risk factor for weight loss, muscle mass loss, and dysphagia, and chemoradiation may be an independent factor [99]. The gold standard for assessing swallowing function is videoendoscopic evaluation of swallowing or swallowing videofluorography [100]. Recently, however, a simple screening method using questionnaires has been developed, and the Eating Assessment Tool-10 (EAT-10) has a cutoff value (19 points) for discriminating dysphagia in patients with head and neck cancer, making it highly useful [100]. Immediately after surgery, oral cancer patients are mainly nourished by tube feeding through nasal catheters. Still, it has been found that patients gradually recover from surgery-induced dysphagia in about 3 months [101]. Although there is concern that parenteral intake during this time may increase a patient’s dependence on tube feeding, a randomized controlled trial concluded that it does not increase the rate of long-term tube dependence after 4 months of treatment, so that it can be used as needed [102]. Almost all patients after oral cancer surgery experience some degree of dysphagia [103]. Unlike dysphagia due to progressive psychiatric illness or cerebrovascular disease without anatomical changes, dysphagia in patients with oral cancer is associated with compensatory behavior. Patients attempt to use their altered mouths in creative ways [103]. Understanding this compensatory behavior and seeking a new swallowing pattern for patients is generally the treatment strategy for dysphagia. The role of rehabilitation and swallowing therapy for patients after oral cancer treatment is to (1) search for safe swallowing strategies with an emphasis on avoiding aspiration, (2) search for therapeutic postures and exercises that may improve swallowing function over time, and (3) modify the diet to ensure safe and adequate oral intake [104]. In recent years, the earlier the timing of rehabilitation intervention, the better, because the usefulness of early rehabilitation from preoperative to immediately after surgery has been reported [105,106,107]. The primary techniques used in rehabilitation are (1) postural techniques, (2) sensory techniques, (3) motor exercises, (4) swallowing maneuvers, and (5) dietary modifications [108]. Specifically, these include changing body position to maximize swallowing function and minimize aspiration, techniques to alter the pressure, taste, and temperature, changing the firmness, placement, and size of the bolus, and increasing the strength, mobility, and durability of the swallowing structures [108,109]. Swallowing maneuvers include supraglottic swallowing, effortful swallowing, and the Mendelssohn maneuver (Figure 6A) [110]. Some results have been obtained in the range of motion training of the tongue and resistance exercises [111,112]. In addition, neuromuscular electrical stimulation has recently been used as an adjunct to swallowing therapy and has proven useful in meta-analyses [108,113,114]. However, a palatal augmentation prosthesis (PAP) can be useful for patients with significant loss of tongue function and articulation after glossectomy [115]. The aim is to reduce the free space between the roof and floor of the oral cavity, to permit stronger lingual propulsion during oral deglutition and better linguopalatal contact during articulation [115]. An observational study using the PAP in 20 post-treatment head and neck cancer patients reported improved oral food transport [116]. In addition, a meta-analysis examining the efficacy of the PAP in patients who have undergone palate surgery demonstrated its effectiveness; therefore, the active use of the PAP is recommended [117]. However, despite the proven efficacy of each of the above intervention methods, there is no standard dysphagia treatment for oral cancer patients, and there is a lack of evidence to support specific protocols; therefore, we are forced to intervene based on “typical” practices and empirical evidence [118]. There is also a lack of evidence on exercise load in rehabilitation, and adherence and compliance to exercise is a major issue, as it has been reported that exercise adherence is low and dropout due to fatigue is particularly high when intense exercise load is used [119,120]. Therefore, consideration should be given to the exercise load, such as setting the goal at approximately 60% of the maximum load [121].

### 3.2. Trismus

Trismus, a narrowing of the distance between the upper and lower incisors, occurs as a complication of surgical and radiation therapy for oral cancer [122,123]. Early manual and mechanical opening training are important for trismus and have been shown to be successful in preventive interventions (Figure 6B) [107,124]. A randomized controlled trial of prophylactic swallowing exercises, including mouth opening training, for patients with head and neck cancer, reported significant differences in the amount of mouth opening at 3 to 6 months [125]. However, due to the long course of trismus, the importance of post-discharge exercises, and the proven effectiveness of follow-up in intervention studies with ongoing follow-up of trismus training, it is advisable to guide all stakeholders related to the patient’s symptoms [126].

### 3.3. Speech Problem

Speech problems occur in patients whose oral anatomy has been altered by surgical treatment, but recent advances in reconstructive surgery have alleviated the symptoms [25]. However, in cases where reconstructive surgery alone does not improve disability, the PAP has effectively treated speech problems [115]. A meta-analysis that examined the effectiveness of the PAP on speech problems reported that the PAP was effective in improving pronunciation [117]. In particular, according to a study that examined the detailed effects of the PAP, the PAP makes a significant contribution to the improvement of consonants [116]. However, there are few reports on PAP morphology, and prosthodontists and speech pathologists need to utilize palatograms to determine PAP morphology, based on resected tongue morphology and reconstructed flap morphology [127].

### 3.4. Taste Loss

It has been reported that damage to the surrounding tissues during radiation therapy for oral cancer can cause taste loss [128]. This is thought to be due to the continued natural death of taste cells and temporary interruption of cell replacement, which is the cause of post-irradiation taste disorder [129]. In addition, xerostomia is known to develop at a high rate after radiotherapy for oral cancer and serves as an accelerator for taste disorders [130]. Taste loss is a sensory disorder that is easily overlooked and neglected by health care providers because the patient’s complaint is often noted but should always be attended to because it can lead to weight loss in patients [131]. It has been reported that four basic tastes change during radiotherapy in patients with head and neck cancer. A recent study confirmed that umami decreased during the third week after radiotherapy and then recovered as early as the eighth week. Therefore, an intervention method that effectively utilizes umami is expected to be developed [132]. Although no other intervention studies have proven effective means of improving taste loss, it is preferable to adopt compensatory methods that are in line with the country’s culture, since food culture has a significant impact on taste loss.

### 3.5. Peripheral Neuropathy of the Oral Cavity

Chemotherapy-induced peripheral neuropathy (CIPN) associated with chemotherapy has been widely reported in patients with head and neck cancer. Still, it has also been reported that patients with head and neck cancer have sensory dysfunction from chemotherapy before receiving treatment [133]. It has been suggested that cancer itself may alter peripheral nerve function and contribute to the development of CIPN [133]. Many studies have reported that peripheral neuropathy occurs with taxane anticancer drugs, but there have been few studies on peripheral neuropathy in the oral cavity [134]. One of the few studies that evaluated oral CIPN in patients with head and neck cancer reported that chemoradiotherapy, including surgery, can cause oral CIPN. Still, the details are not clear, and the treatment of oral CIPN is unknown.

### 3.6. Esthetic Disorder

Esthetic disorder refers to the morphological changes in the oral cavity or perioral region caused by surgical treatment and is regarded as an unmet clinical need for oral cancer treatment [135]. However, patients who have lost both oral and maxillofacial tissues need rehabilitation management to improve esthetics and restore oral function [136]. The limited evidence suggests that it is important to provide prosthetic treatment and reconstructive surgery with flaps for patients who have undergone maxillary and mandibular surgery, through the collaboration of prosthodontists, plastic surgeons, and oral surgeons [137,138].

## 4. Psychosocial Issues and Quality of Life

The assessment of QoL for patients with oral cancer is important, and many questionnaires (UW-QOL, MDADI, and EORTC QLQ-H&N35) have been developed and used in clinical and research settings [139,140,141]. Oral cancer treatment is known to be one of the cancer treatments with the poorest prognosis and most degraded quality of life, especially because it impairs patients’ social and physical functions [142]. The high rate of suicide among oral cancer patients is particularly striking due to their reduced QoL, and it has been reported that they are 12 times more likely to commit suicide than the general American population [142]. Therefore, a multidisciplinary team of experts is needed, and it is important to measure QoL continuously. It should always be noted that many problems can be attributed to oral rehabilitation [143,144].

## 5. Limitation

Since this paper was written using the narrative review method, a bias may exist in the article selection. The narrative review methodology was adopted due to large uncertainty about oral function after oral cancer treatment, and the current scattered literature. Once additional literature on oral function after oral cancer treatment is generated, systematic reviews will be warranted.

## 6. Conclusions and Perspective

Although the future development of new oral cancer treatments (the development of surgical methods, new anticancer drugs, and improved accuracy of radiotherapy) is expected, surgery is often the first priority when considering survival rates and will continue to be for some time to come. Therefore, there are high hopes for developing and advancing supportive care and rehabilitation methods to improve the quality of oral cancer treatment (Table 1). As shown in Table 1, all stakeholders must be involved with the patient, in addition to the oral and maxillofacial surgeons, and the head and neck surgeons from the clinical practice. At the same time, this means that an advanced team approach involving multiple professionals, such as oral and maxillofacial surgeons, dentists, dental hygienists, physiatrists, speech pathologists, and nurses is necessary. As for oral health care for oral cancer patients, systems have been rapidly established in Japan over the past few years, and the importance of oral health care is being recognized. With regard to rehabilitation of oral cancer, although there is little evidence, a certain amount of progress has been made in the development of treatment methods tailored to individual symptoms. However, because oral cancer is rare, there is little evidence, and it is not systematized, and we must rely on empiric therapies. The first challenge is to change the current situation and create a consensus-based flow of supportive care and rehabilitation in oral cancer treatment.

## Figures and Tables

**Table 1:** Specific interventions for oral health management in oral cancer treatment.

Oral Health Management			
Oral Function Management	Oral Hygiene Management	Oral Care	
		Oral Cleaning	Meal Preparation
Dental caries treatment (D) Root canal treatment (D) Periodontal treatment (D/DH) Tooth extraction (D) Prosthodontic treatment (D) Swallowing rehabilitation (D/DH)	Biofilm removal (DH) Cleaning of the oral mucosa (DH) Dental scaling (DH)	Oral cleaning (N/T/F) Toothbrush storage (N/T/F) Denture storage (N/T/F)	Swallowing exercise (N/T/F) Salivary gland massage (N/T/F) Posture adjustment (N/T/F) Assistance with eating (N/F)
Environmental settings for oral health management (All) Providing knowledge (D/DH/N) Behavior modification (D/DH) Instruction in self-care for patients and families (D/DH) Approaches to oral health related-self-efficacy and literacy (D/DH)			

(D): dentist, (DH): dental hygienist, (N): nurse, (T): themselves, (F): their family, (All): All stakeholders related to the patient.

## Data Availability

Not applicable.

## References

[1] Warnakulasuriya S. (2009). Global epidemiology of oral and oropharyngeal cancer. Oral Oncol..

[2] Petersen P.E. (2003). The World Oral Health Report 2003: Continuous improvement of oral health in the 21st century—The approach of the WHO Global Oral Health Programme. Community Dent. Oral Epidemiol..

[3] Sarode G., Maniyar N., Sarode S.C., Jafer M., Patil S., Awan K.H. (2020). Epidemiologic aspects of oral cancer. Dis. Mon..

[4] Crile G. (1987). Landmark article 1 December 1906: Excision of cancer of the head and neck. With special reference to the plan of dissection based on one hundred and thirty-two operations. By George Crile. JAMA.

[5] Inchingolo F., Santacroce L., Ballini A., Topi S., Dipalma G., Haxhirexha K., Bottalico L., Charitos I.A. (2020). Oral Cancer: A Historical Review. Int. J. Environ. Res. Public Health.

[6] Van Baar G.J.C., Forouzanfar T., Liberton N., Winters H.A.H., Leusink F.K.J. (2018). Accuracy of computer-assisted surgery in mandibular reconstruction: A systematic review. Oral Oncol..

[7] Blanchard P., Belkhir F., Temam S., El Khoury C., De Felice F., Casiraghi O., Patrikidou A., Mirghani H., Levy A., Even C. (2017). Outcomes and prognostic factors for squamous cell carcinoma of the oral tongue in young adults: A single-institution case-matched analysis. Eur. Arch. Otorhinolaryngol..

[8] Brennan P.A., Bradley K.L., Brands M. (2017). Intensity-modulated radiotherapy in head and neck cancer—An update for oral and maxillofacial surgeons. Br. J. Oral Maxillofac. Surg..

[9] Patil V.M., Noronha V., Joshi A., Abhyankar A., Menon N., Dhumal S., Prabhash K. (2020). Beyond conventional chemotherapy, targeted therapy and immunotherapy in squamous cell cancer of the oral cavity. Oral Oncol..

[10] Xu Y., Wen N., Sonis S.T., Villa A. (2021). Oral side effects of immune checkpoint inhibitor therapy (ICIT): An analysis of 4683 patients receiving ICIT for malignancies at Massachusetts General Hospital, Brigham & Women’s Hospital, and the Dana-Farber Cancer Institute, 2011 to 2019. Cancer.

[11] Moore K.A., Ford P.J., Farah C.S. (2014). Support needs and quality of life in oral cancer: A systematic review. Int. J. Dent. Hyg..

[12] Friedlander A.H., Rosenbluth S.C., Rubin R.T. (2012). The adult suicide-prone patient: A review of the medical literature and implications for oral and maxillofacial surgeons. J. Oral Maxillofac. Surg..

[13] Twigg J.A., Anderson J.M., Humphris G., Nixon I., Rogers S.N., Kanatas A. (2020). Best practice in reducing the suicide risk in head and neck cancer patients: A structured review. Br. J. Oral Maxillofac. Surg..

[14] Licitra L., Keilholz U., Tahara M., Lin J.C., Chomette P., Ceruse P., Harrington K., Mesia R. (2016). Evaluation of the benefit and use of multidisciplinary teams in the treatment of head and neck cancer. Oral Oncol..

[15] De Felice F., de Vincentiis M., Valentini V., Musio D., Mezi S., Lo Mele L., Terenzi V., D’Aguanno V., Cassoni A., Di Brino M. (2017). Follow-up program in head and neck cancer. Crit. Rev. Oncol. Hematol..

[16] Ohara Y., Iwasaki M., Motokawa K., Hirano H. (2021). Preliminary investigation of family caregiver burden and oral care provided to homebound older patients. Clin. Exp. Dent. Res..

[17] Wong F.M.F., Ng Y.T.Y., Leung W.K. (2019). Oral Health and Its Associated Factors Among Older Institutionalized Residents—A Systematic Review. Int. J. Environ. Res. Public Health.

[18] Matsuda Y., Karino M., Okuma S., Ikebuchi K., Takeda M., Kanno T. (2020). Proposal of Dental Hygiene Diagnosis for Cancer Patients Based on Dental Hygiene Process of Care in Acute Care Hospitals: A Narrative Review. Healthcare.

[19] Itohara C., Matsuda Y., Sukegawa-Takahashi Y., Sukegawa S., Furuki Y., Kanno T. (2020). Relationship between Oral Health Status and Postoperative Fever among Patients with Lung Cancer Treated by Surgery: A Retrospective Cohort Study. Healthcare.

[20] Minakuchi S., Tsuga K., Ikebe K., Ueda T., Tamura F., Nagao K., Furuya J., Matsuo K., Yamamoto K., Kanazawa M. (2018). Oral hypofunction in the older population: Position paper of the Japanese Society of Gerodontology in 2016. Gerodontology.

[21] Kageyama S., Nagao Y., Ma J., Asakawa M., Yoshida R., Takeshita T., Hirosue A., Yamashita Y., Nakayama H. (2020). Compositional Shift of Oral Microbiota Following Surgical Resection of Tongue Cancer. Front. Cell. Infect. Microbiol..

[22] Yamamoto T., Ueta E., Kamatani T., Osaki T. (2005). DNA identification of the pathogen of candidal aspiration pneumonia induced in the course of oral cancer therapy. J. Med. Microbiol..

[23] Kim R.H., Yang P., Sung E.C. (2016). Managing Intraoral Lesions in Oral Cancer Patients in a General Dental Practice: An Overview. J. Calif. Dent. Assoc..

[24] Villa A., Akintoye S.O. (2018). Dental Management of Patients Who Have Undergone Oral Cancer Therapy. Dent. Clin. N. Am..

[25] Davudov M.M., Harirchi I., Arabkheradmand A., Garajei A., Mahmudzadeh H., Shirkhoda M., Motiee-Langroudi M., Mirzajani Z., Zebardast J., Montazeri A. (2019). Evaluation of quality of life in patients with oral cancer after mandibular resection: Comparing no reconstruction, reconstruction with plate, and reconstruction with flap. Medicine.

[26] Ge X., Liao Z., Yuan J., Mao D., Li Y., Yu E., Wang X., Ding Z. (2020). Radiotherapy-related quality of life in patients with head and neck cancers: A meta-analysis. Support. Care Cancer.

[27] Wolff A., Joshi R.K., Ekstrom J., Aframian D., Pedersen A.M., Proctor G., Narayana N., Villa A., Sia Y.W., Aliko A. (2017). A Guide to Medications Inducing Salivary Gland Dysfunction, Xerostomia, and Subjective Sialorrhea: A Systematic Review Sponsored by the World Workshop on Oral Medicine VI. Drugs R&d.

[28] Furness S., Bryan G., McMillan R., Birchenough S., Worthington H.V. (2013). Interventions for the management of dry mouth: Non-pharmacological interventions. Cochrane Database Syst. Rev..

[29] Zheng K., Liao Z., Yoda N., Fang J., Chen J., Zhang Z., Zhong J., Peck C., Sasaki K., Swain M.V. (2019). Investigation on masticatory muscular functionality following oral reconstruction—An inverse identification approach. J. Biomech..

[30] Ono T., Kohda H., Hori K., Nokubi T. (2007). Masticatory performance in postmaxillectomy patients with edentulous maxillae fitted with obturator prostheses. Int. J. Prosthodont..

[31] Curtis D.A., Plesh O., Hannam A.G., Sharma A., Curtis T.A. (1999). Modeling of jaw biomechanics in the reconstructed mandibulectomy patient. J. Prosthet. Dent..

[32] Huang Y.F., Chang C.T., Muo C.H., Hong H.H., Shen Y.F., Lin C.C., Liu S.P. (2018). The association of variables of fibular reconstructed mandible and bite force in oral cancer patients with dental implant rehabilitation. J. Craniomaxillofac. Surg..

[33] Fujikawa N., Ogino Y., Koga S., Ueno M., Moroi R., Koyano K. (2021). Validation of masticatory function and related factors in maxillectomy patients based on the concept of “oral hypofunction”: A retrospective cross-sectional study. J. Prosthodont. Res..

[34] Ji Y.B., Cho Y.H., Song C.M., Kim Y.H., Kim J.T., Ahn H.C., Tae K. (2017). Long-term functional outcomes after resection of tongue cancer: Determining the optimal reconstruction method. Eur. Arch. Otorhinolaryngol..

[35] De Groot R.J., Merkx M.A.W., Hamann M.N.S., Brand H.S., de Haan A.F.J., Rosenberg A., Speksnijder C.M. (2020). Tongue function and its influence on masticatory performance in patients treated for oral cancer: A five-year prospective study. Support. Care Cancer.

[36] De Vicente J.C., de Villalain L., Torre A., Pena I. (2008). Microvascular free tissue transfer for tongue reconstruction after hemiglossectomy: A functional assessment of radial forearm versus anterolateral thigh flap. J. Oral Maxillofac. Surg..

[37] Wang L., Liu K., Shao Z., Shang Z.J. (2016). Individual design of the anterolateral thigh flap for functional reconstruction after hemiglossectomy: Experience with 238 patients. Int. J. Oral Maxillofac. Surg..

[38] Xing F., Stone M., Prince J.L., Liu X., Fakhri G.E., Woo J. (2021). Floor-of-the-Mouth Muscle Function Analysis Using Dynamic Magnetic Resonance Imaging. Proc. SPIE Int. Soc. Opt. Eng..

[39] Ogino Y., Fujikawa N., Koga S., Moroi R., Koyano K. (2021). A retrospective cross-sectional analysis of swallowing and tongue functions in maxillectomy patients. Support. Care Cancer.

[40] Robbins J., Gangnon R.E., Theis S.M., Kays S.A., Hewitt A.L., Hind J.A. (2005). The effects of lingual exercise on swallowing in older adults. J. Am. Geriatr. Soc..

[41] Tashimo Y., Ihara Y., Yuasa K., Nozue S., Saito Y., Katsuta H., Shimane T., Takahashi K. (2019). Acute Stage Longitudinal Change of Quality of Life from Pre- to 3 Months after Surgical Treatment in Head and Neck Cancer Patients. Asian Pac. J. Cancer Prev..

[42] Rogus-Pulia N.M., Larson C., Mittal B.B., Pierce M., Zecker S., Kennelty K., Kind A., Connor N.P. (2016). Effects of Change in Tongue Pressure and Salivary Flow Rate on Swallow Efficiency Following Chemoradiation Treatment for Head and Neck Cancer. Dysphagia.

[43] Hasegawa Y., Sugahara K., Fukuoka T., Saito S., Sakuramoto A., Horii N., Sano S., Hasegawa K., Nakao Y., Nanto T. (2017). Change in tongue pressure in patients with head and neck cancer after surgical resection. Odontology.

[44] Namaki S., Tanaka T., Hara Y., Ohki H., Shinohara M., Yonhehara Y. (2011). Videofluorographic evaluation of dysphagia before and after modification of the flap and scar in patients with oral cancer. J. Plast. Surg. Hand Surg..

[45] Namiki C., Hara K., Tohara H., Kobayashi K., Chantaramanee A., Nakagawa K., Saitou T., Yamaguchi K., Yoshimi K., Nakane A. (2019). Tongue-pressure resistance training improves tongue and suprahyoid muscle functions simultaneously. Clin. Interv. Aging.

[46] Speksnijder C.M., van der Bilt A., Abbink J.H., Merkx M.A., Koole R. (2011). Mastication in patients treated for malignancies in tongue and/or floor of mouth: A 1-year prospective study. Head Neck.

[47] Hahn T.R., Kruskemper G., Enkling N., Kubler N.R. (2007). On quality of life after surgical therapy of oral cancer—A retrospective multi-center study: The connection between dedentition, denture, quality of life, and dysphagia, and the resulting rehabilitation schemes. Mund Kiefer Gesichtschirurgie.

[48] Vijayaraghavan N.V., Ramesh G., Thareja A., Patil S. (2015). Masticatory efficiency after rehabilitation of acquired maxillary and mandibular defects. Indian J. Dent..

[49] De Groot R.J., Wetzels J.W., Merkx M.A.W., Rosenberg A., de Haan A.F.J., van der Bilt A., Abbink J.H., Speksnijder C.M. (2019). Masticatory function and related factors after oral oncological treatment: A 5-year prospective study. Head Neck.

[50] Friedlander A.H., Tajima T., Kawakami K.T., Wang M.B., Tomlinson J. (2008). The relationship between measures of nutritional status and masticatory function in untreated patients with head and neck cancer. J. Oral Maxillofac. Surg..

[51] Hagio M., Ishizaki K., Ryu M., Nomura T., Takano N., Sakurai K. (2018). Maxillofacial prosthetic treatment factors affecting oral health-related quality of life after surgery for patients with oral cancer. J. Prosthet. Dent..

[52] Siddall K.Z., Rogers S.N., Butterworth C.J. (2012). The prosthodontic pathway of the oral cancer patient. Dent. Update.

[53] Said M.M., Otomaru T., Sumita Y., Leung K.C.M., Khan Z., Taniguchi H. (2017). Systematic review of literature: Functional outcomes of implant-prosthetic treatment in patients with surgical resection for oral cavity tumors. J. Investig. Clin. Dent..

[54] Murase R., Ishikawa A., Sumida T., Shinohara K., Nakashiro K., Hamakawa H. (2016). Objective validity of an implant-retained overdenture with a ball attachment system after marginal mandibulectomy. Br. J. Oral Maxillofac. Surg..

[55] Huang Y.F., Liu S.P., Muo C.H., Chang C.T. (2020). Prosthetic design related to peri-implant bone resorption in microvascular free fibular flap among patients with oral cancer: A retrospective clinical study. J. Prosthet. Dent..

[56] Morais M.O., Elias M.R., Leles C.R., Dourado Pinezi J.C., Mendonca E.F. (2016). The effect of preventive oral care on treatment outcomes of a cohort of oral cancer patients. Support. Care Cancer.

[57] Yamada S.I., Soutome S., Hasegawa T., Tojyo I., Nakahara H., Kawakami M., Hirose M., Fujita S., Komori T., Kirita T. (2020). A multicenter retrospective investigation on the efficacy of perioperative oral management in cancer patients. Medicine.

[58] Epstein J.B., Barasch A. (2018). Oral and Dental Health in Head and Neck Cancer Patients. Multidisciplinary Care of the Head and Neck Cancer Patient.

[59] Aoki T., Kudo M., Endo M., Nakayama Y., Amano A., Naito M., Ota Y. (2019). Inter-rater reliability of the Oral Assessment Guide for oral cancer patients between nurses and dental hygienists: The difficulties in objectively assessing oral health. Support. Care Cancer.

[60] Bohm N., Karlsson C., Skoogh Andersson J., Almstahl A. (2020). Variations in odontological care routines for patients undergoing treatment for head and neck cancer in county councils/regions of Sweden. Clin. Exp. Dent. Res..

[61] Epstein J.B., Smith D.K., Villines D., Parker I., Hameroff J., Hill B.R., Murphy B.A. (2018). Patterns of oral and dental care education and utilization in head and neck cancer patients. Support. Care Cancer.

[62] Deng J., Jackson L., Epstein J.B., Migliorati C.A., Murphy B.A. (2015). Dental demineralization and caries in patients with head and neck cancer. Oral Oncol..

[63] Cai J., Palamara J., Manton D.J., Burrow M.F. (2018). Status and progress of treatment methods for root caries in the last decade: A literature review. Aust. Dent. J..

[64] Papas A., Russell D., Singh M., Kent R., Triol C., Winston A. (2008). Caries clinical trial of a remineralising toothpaste in radiation patients. Gerodontology.

[65] Epstein J.B., Stevenson-Moore P. (2001). Periodontal disease and periodontal management in patients with cancer. Oral Oncol..

[66] Sroussi H.Y., Epstein J.B., Bensadoun R.J., Saunders D.P., Lalla R.V., Migliorati C.A., Heaivilin N., Zumsteg Z.S. (2017). Common oral complications of head and neck cancer radiation therapy: Mucositis, infections, saliva change, fibrosis, sensory dysfunctions, dental caries, periodontal disease, and osteoradionecrosis. Cancer Med..

[67] Bueno A.C., Ferreira R.C., Barbosa F.I., Jham B.C., Magalhaes C.S., Moreira A.N. (2013). Periodontal care in patients undergoing radiotherapy for head and neck cancer. Support. Care Cancer.

[68] Bossi P., Numico G., De Santis V., Ruo Redda M.G., Reali A., Belgioia L., Cossu Rocca M., Orlandi E., Airoldi M., Bacigalupo A. (2014). Prevention and treatment of oral mucositis in patients with head and neck cancer treated with (chemo) radiation: Report of an Italian survey. Support. Care Cancer.

[69] Sonis S.T. (2004). A biological approach to mucositis. J. Support Oncol..

[70] Kawashita Y., Koyama Y., Kurita H., Otsuru M., Ota Y., Okura M., Horie A., Sekiya H., Umeda M. (2019). Effectiveness of a comprehensive oral management protocol for the prevention of severe oral mucositis in patients receiving radiotherapy with or without chemotherapy for oral cancer: A multicentre, phase II, randomized controlled trial. Int. J. Oral Maxillofac. Surg..

[71] Liu Y.J., Zhu G.P., Guan X.Y. (2012). Comparison of the NCI-CTCAE version 4.0 and version 3.0 in assessing chemoradiation-induced oral mucositis for locally advanced nasopharyngeal carcinoma. Oral Oncol..

[72] Hong C.H.L., Gueiros L.A., Fulton J.S., Cheng K.K.F., Kandwal A., Galiti D., Fall-Dickson J.M., Johansen J., Ameringer S., Kataoka T. (2019). Systematic review of basic oral care for the management of oral mucositis in cancer patients and clinical practice guidelines. Support. Care Cancer.

[73] Iwamoto M., Morikawa T., Narita M., Shibahara T., Katakura A. (2020). Investigation of Surgical Site Infections and Bacteria Detected Following Neck Dissection in Patients with Oral Cancer. Bull. Tokyo Dent. Coll..

[74] Shigeishi H., Ohta K., Fujimoto S., Nakagawa T., Mizuta K., Ono S., Shimasue H., Ninomiya Y., Higashikawa K., Tada M. (2016). Preoperative oral health care reduces postoperative inflammation and complications in oral cancer patients. Exp. Ther. Med..

[75] Funahara M., Yanamoto S., Ueda M., Suzuki T., Ota Y., Nishimaki F., Kurita H., Yamakawa N., Kirita T., Okura M. (2017). Prevention of surgical site infection after oral cancer surgery by topical tetracycline: Results of a multicenter randomized control trial. Medicine.

[76] Funahara M., Hayashida S., Sakamoto Y., Yanamoto S., Kosai K., Yanagihara K., Umeda M. (2015). Efficacy of topical antibiotic administration on the inhibition of perioperative oral bacterial growth in oral cancer patients: A preliminary study. Int. J. Oral Maxillofac. Surg..

[77] Liao P.H., Chu C.H., Hung Y.M., Tang P.L., Kuo T.J. (2021). Tumor subsites and risk of osteoradionecrosis of the jaw in patients with oral cavity cancer: A national-based cohort study. Eur. Arch. Otorhinolaryngol..

[78] Ranta P., Kyto E., Nissi L., Kinnunen I., Vahlberg T., Minn H., Haapio E., Nelimarkka L., Irjala H. (2022). Dysphagia, hypothyroidism, and osteoradionecrosis after radiation therapy for head and neck cancer. Laryngoscope Investig. Otolaryngol..

[79] Kubota H., Miyawaki D., Mukumoto N., Ishihara T., Matsumura M., Hasegawa T., Akashi M., Kiyota N., Shinomiya H., Teshima M. (2021). Risk factors for osteoradionecrosis of the jaw in patients with head and neck squamous cell carcinoma. Radiat. Oncol..

[80] Kuhnt T., Stang A., Wienke A., Vordermark D., Schweyen R., Hey J. (2016). Potential risk factors for jaw osteoradionecrosis after radiotherapy for head and neck cancer. Radiat. Oncol..

[81] Chang C.T., Liu S.P., Muo C.H., Tsai C.H., Huang Y.F. (2017). Dental Prophylaxis and Osteoradionecrosis: A Population-Based Study. J. Dent. Res..

[82] Manne S., Hudson S., Frederick S., Mitarotondo A., Baredes S., Kalyoussef E., Ohman-Strickland P., Kashy D.A. (2020). e-Health self-management intervention for oral and oropharyngeal cancer survivors: Design and single-arm pilot study of empowered survivor. Head Neck.

[83] Ohrn K.E., Sjoden P.O. (2003). Experiences of oral care in patients with haematological malignancies or head and neck cancer. Eur. J. Cancer Care.

[84] Matsuda Y., Karino M., Kanno T. (2021). Development and Validation of the Oral Health-Related Self-Efficacy Scale for Cancer Patients. J. Cancer Educ..

[85] Ramandeep G., Arshdeep S., Vinod K., Parampreet P. (2014). Oral health literacy among clients visiting a rural dental college in North India-a cross-sectional study. Ethiop. J. Health Sci..

[86] Daniel B.T., Damato K.L., Johnson J. (2004). Educational issues in oral care. Semin. Oncol. Nurs..

[87] Matear D.W. (1999). Demonstrating the need for oral health education in geriatric institutions. Probe.

[88] Strauss R.P. (1988). The patient with cancer: Social and clinical perspectives for the dentist. Spec. Care Dent..

[89] Badr H., Yeung C., Lewis M.A., Milbury K., Redd W.H. (2015). An observational study of social control, mood, and self-efficacy in couples during treatment for head and neck cancer. Psychol. Health.

[90] Kristanti M.S., Setiyarini S., Effendy C. (2017). Enhancing the quality of life for palliative care cancer patients in Indonesia through family caregivers: A pilot study of basic skills training. BMC Palliat. Care.

[91] Igarashi A., Morita T., Miyashita M., Kiyohara E., Inoue S. (2010). Changes in medical and nursing care after admission to palliative care units: A potential method for improving regional palliative care. Support. Care Cancer.

[92] Sen S., Priyadarshini S.R., Sahoo P.K., Dutta A., Singh A.K., Kumar U. (2020). Palliative oral care in patients undergoing radiotherapy: Integrated review. J. Fam. Med. Prim. Care.

[93] Stokman M.A., Vissink A., Spijkervet F.K. (2008). Foci of infection and oral supportive care in cancer patients. Nederlands Tijdschrift Tandheelkunde.

[94] Pace-Balzan A., Shaw R.J., Butterworth C. (2011). Oral rehabilitation following treatment for oral cancer. Periodontol. 2000.

[95] Kahrilas P.J., Logemann J.A., Krugler C., Flanagan E. (1991). Volitional augmentation of upper esophageal sphincter opening during swallowing. Am. J. Physiol..

[96] Palmer J.B. (1998). Bolus aggregation in the oropharynx does not depend on gravity. Arch. Phys. Med. Rehabil..

[97] Ohkoshi A., Ogawa T., Nakanome A., Ishida E., Ishii R., Kato K., Katori Y. (2018). Predictors of chewing and swallowing disorders after surgery for locally advanced oral cancer with free flap reconstruction: A prospective, observational study. Surg. Oncol..

[98] Depeyre A., Pereira B., Pham-Dang N., Barthelemy I., Hennequin M. (2020). Impairments in Food Oral Processing in Patients Treated for Tongue Cancer. Dysphagia.

[99] Kagifuku Y., Tohara H., Wakasugi Y., Susa C., Nakane A., Toyoshima M., Nakakuki K., Kabasawa Y., Harada H., Minakuchi S. (2020). What Factors Affect Changes in Body Composition and Swallowing Function in Patients Hospitalized for Oral Cancer Surgery?. Clin. Interv. Aging.

[100] Florie M., Pilz W., Kremer B., Verhees F., Waltman G., Winkens B., Winter N., Baijens L. (2021). EAT-10 Scores and Fiberoptic Endoscopic Evaluation of Swallowing in Head and Neck Cancer Patients. Laryngoscope.

[101] Ohkoshi A., Kato K., Ogawa T., Nakanome A., Ishii R., Katori Y. (2020). Improvement of a delayed swallowing reflex following treatment for advanced head and neck cancer. Cancers Head Neck.

[102] Brown T., Banks M., Hughes B.G.M., Lin C., Kenny L.M., Bauer J.D. (2017). Impact of early prophylactic feeding on long term tube dependency outcomes in patients with head and neck cancer. Oral Oncol..

[103] Halczy-Kowalik L., Wiktor A., Rzewuska A., Kowalczyk R., Wysocki R., Posio V. (2015). Compensatory Mechanisms in Patients After a Partial or Total Glossectomy due to Oral Cancer. Dysphagia.

[104] Murphy B.A., Gilbert J. (2009). Dysphagia in head and neck cancer patients treated with radiation: Assessment, sequelae, and rehabilitation. Semin. Radiat. Oncol..

[105] Bschorer M., Schneider D., Hennig M., Frank B., Schon G., Heiland M., Bschorer R. (2018). Early intensive rehabilitation after oral cancer treatment. J. Craniomaxillofac. Surg..

[106] Van Daele D.J., Langmore S.E., Krisciunas G.P., Lazarus C.L., Pauloski B.R., McCulloch T.M., Gramigna G.D., Messing B.P., Wagner C.W., Mott S.L. (2019). The impact of time after radiation treatment on dysphagia in patients with head and neck cancer enrolled in a swallowing therapy program. Head Neck.

[107] Van der Molen L., van Rossum M.A., Burkhead L.M., Smeele L.E., Rasch C.R., Hilgers F.J. (2011). A randomized preventive rehabilitation trial in advanced head and neck cancer patients treated with chemoradiotherapy: Feasibility, compliance, and short-term effects. Dysphagia.

[108] Mittal B.B., Pauloski B.R., Haraf D.J., Pelzer H.J., Argiris A., Vokes E.E., Rademaker A., Logemann J.A. (2003). Swallowing dysfunction—Preventative and rehabilitation strategies in patients with head-and-neck cancers treated with surgery, radiotherapy, and chemotherapy: A critical review. Int. J. Radiat. Oncol. Biol. Phys..

[109] Martin-Harris B., Brodsky M.B., Michel Y., Ford C.L., Walters B., Heffner J. (2005). Breathing and swallowing dynamics across the adult lifespan. Arch. Otolaryngol. Head Neck Surg..

[110] Alexander M.S., Edelman G.M., Man K., Randall C.J. (1989). Dysphagia after fast neutron therapy to the head and neck. Br. J. Radiol..

[111] Logemann J.A., Pauloski B.R., Rademaker A.W., Colangelo L.A. (1997). Speech and swallowing rehabilitation for head and neck cancer patients. Oncology.

[112] Jordan K. (1979). Rehabilitation of the patient with dysphagia. Ear Nose Throat J..

[113] Langmore S.E., McCulloch T.M., Krisciunas G.P., Lazarus C.L., Van Daele D.J., Pauloski B.R., Rybin D., Doros G. (2016). Efficacy of electrical stimulation and exercise for dysphagia in patients with head and neck cancer: A randomized clinical trial. Head Neck.

[114] Ryu J.S., Kang J.Y., Park J.Y., Nam S.Y., Choi S.H., Roh J.L., Kim S.Y., Choi K.H. (2009). The effect of electrical stimulation therapy on dysphagia following treatment for head and neck cancer. Oral Oncol..

[115] Robbins K.T., Bowman J.B., Jacob R.F. (1987). Postglossectomy deglutitory and articulatory rehabilitation with palatal augmentation prostheses. Arch. Otolaryngol. Head Neck Surg..

[116] Lofhede H., Wertsen M., Havstam C. (2020). Palatal augmentation prostheses in individuals treated for head and neck cancer: Effects on speech and oral transport. Head Neck.

[117] Marunick M., Tselios N. (2004). The efficacy of palatal augmentation prostheses for speech and swallowing in patients undergoing glossectomy: A review of the literature. J. Prosthet. Dent..

[118] Krisciunas G.P., Sokoloff W., Stepas K., Langmore S.E. (2012). Survey of usual practice: Dysphagia therapy in head and neck cancer patients. Dysphagia.

[119] Mortensen H.R., Jensen K., Aksglaede K., Lambertsen K., Eriksen E., Grau C. (2015). Prophylactic Swallowing Exercises in Head and Neck Cancer Radiotherapy. Dysphagia.

[120] Krisciunas G.P., Castellano K., McCulloch T.M., Lazarus C.L., Pauloski B.R., Meyer T.K., Graner D., Van Daele D.J., Silbergleit A.K., Crujido L.R. (2017). Impact of Compliance on Dysphagia Rehabilitation in Head and Neck Cancer Patients: Results from a Multi-center Clinical Trial. Dysphagia.

[121] Van Nuffelen G., Van den Steen L., Vanderveken O., Specenier P., Van Laer C., Van Rompaey D., Guns C., Marien S., Peeters M., Van de Heyning P. (2015). Study protocol for a randomized controlled trial: Tongue strengthening exercises in head and neck cancer patients, does exercise load matter?. Trials.

[122] Agarwal P., Shiva Kumar H.R., Rai K.K. (2016). Trismus in oral cancer patients undergoing surgery and radiotherapy. J. Oral Biol. Craniofac. Res..

[123] Wetzels J.W., Merkx M.A., de Haan A.F., Koole R., Speksnijder C.M. (2014). Maximum mouth opening and trismus in 143 patients treated for oral cancer: A 1-year prospective study. Head Neck.

[124] Weber C., Dommerich S., Pau H.W., Kramp B. (2010). Limited mouth opening after primary therapy of head and neck cancer. Oral Maxillofac. Surg..

[125] Messing B.P., Ward E.C., Lazarus C.L., Kim M., Zhou X., Silinonte J., Gold D., Harrer K., Ulmer K., Merritt S. (2017). Prophylactic Swallow Therapy for Patients with Head and Neck Cancer Undergoing Chemoradiotherapy: A Randomized Trial. Dysphagia.

[126] Wang T.J., Su J.H., Leung K.W., Liang S.Y., Wu S.F., Wang H.M. (2019). Effects of a mouth-opening intervention with remote support on adherence, the maximum interincisal opening, and mandibular function of postoperative oral cancer patients: A randomized clinical trial. Eur. J. Oncol. Nurs..

[127] Kikutani T., Tamura F., Nishiwaki K. (2006). Case presentation: Dental treatment with PAP for ALS patient. Int. J. Orofac. Myol..

[128] Dreizen S., Daly T.E., Drane J.B., Brown L.R. (1977). Oral complications of cancer radiotherapy. Postgrad. Med..

[129] Nguyen H.M., Reyland M.E., Barlow L.A. (2012). Mechanisms of taste bud cell loss after head and neck irradiation. J. Neurosci..

[130] Dirix P., Nuyts S., Vander Poorten V., Delaere P., Van den Bogaert W. (2008). The influence of xerostomia after radiotherapy on quality of life: Results of a questionnaire in head and neck cancer. Support. Care Cancer.

[131] Jin S., Lu Q., Jin S., Zhang L., Cui H., Li H. (2018). Relationship between subjective taste alteration and weight loss in head and neck cancer patients treated with radiotherapy: A longitudinal study. Eur. J. Oncol. Nurs..

[132] Yamashita H., Nakagawa K., Hosoi Y., Kurokawa A., Fukuda Y., Matsumoto I., Misaka T., Abe K. (2009). Umami taste dysfunction in patients receiving radiotherapy for head and neck cancer. Oral Oncol..

[133] Roldan C.J., Johnson C., Lee S.O., Peng A., Dougherty P.M., Huh B. (2018). Subclinical Peripheral Neuropathy in Patients with Head and Neck Cancer: A Quantitative Sensory Testing (QST) Study. Pain Physician.

[134] Forastiere A.A. (1994). Current and future trials of Taxol (paclitaxel) in head and neck cancer. Ann. Oncol..

[135] Zhang Q.Z., Chen C., Chang M.B., Shanti R.M., Cannady S.B., O’Malley B.W., Shi S., Le A.D. (2019). Oral Rehabilitation of Patients Sustaining Orofacial Injuries: The UPenn Initiative. Adv. Dent. Res..

[136] Barclay C.W., Foster E.C., Taylor C.L. (2018). Restorative aspects of oral cancer reconstruction. Br. Dent. J..

[137] Abdel Fattah H., Zaghloul A. (2010). Pre-prosthetic surgical alterations in maxillectomy to enhance the prosthetic prognoses as part of rehabilitation of oral cancer patient. J. Egypt. Natl. Cancer Inst..

[138] Attia S., Wiltfang J., Streckbein P., Wilbrand J.F., El Khassawna T., Mausbach K., Howaldt H.P., Schaaf H. (2019). Functional and aesthetic treatment outcomes after immediate jaw reconstruction using a fibula flap and dental implants. J. Craniomaxillofac. Surg..

[139] Singer S., Arraras J.I., Chie W.C., Fisher S.E., Galalae R., Hammerlid E., Nicolatou-Galitis O., Schmalz C., Verdonck-de Leeuw I., Gamper E. (2013). Performance of the EORTC questionnaire for the assessment of quality of life in head and neck cancer patients EORTC QLQ-H&N35: A methodological review. Qual. Life Res..

[140] Villaret A.B., Cappiello J., Piazza C., Pedruzzi B., Nicolai P. (2008). Quality of life in patients treated for cancer of the oral cavity requiring reconstruction: A prospective study. Acta Otorhinolaryngol. Ital..

[141] Matsuda Y., Kanazawa M., Komagamine Y., Yamashiro M., Akifusa S., Minakuchi S. (2018). Reliability and Validity of the MD Anderson Dysphagia Inventory Among Japanese Patients. Dysphagia.

[142] Mahalingam S., Spielmann P. (2019). Quality of Life Outcomes following Treatment of Hypopharyngeal Cancer. Adv. Otorhinolaryngol..

[143] Honkasalo T., Nissinen E. (1988). Determination of phenol sulphotransferase activity by high-performance liquid chromatography. J. Chromatogr..

[144] Petrovic I., Rosen E.B., Matros E., Huryn J.M., Shah J.P. (2018). Oral rehabilitation of the cancer patient: A formidable challenge. J. Surg. Oncol..

